# Influence of cellular models and individual factor in the biological response to chest CT scan exams

**DOI:** 10.1186/s41747-022-00266-0

**Published:** 2022-03-17

**Authors:** Clément Devic, Larry Bodgi, Laurène Sonzogni, Frank Pilleul, Hervé Ribot, Charlotte De Charry, François Le Moigne, Didier Paul, Fanny Carbillet, Mélodie Munier, Nicolas Foray

**Affiliations:** 1 Institut National de la Santé et de la Recherche Médicale, U1296, « Radiations: Defense, Health and Environment », Bât Cheney A 28 Rue Laennec Centre Léon-Bérard, 69008 Lyon, France; 2 Fibermetrix™ SAS, 7 Allée de l’Europe, 67960, Entzheim, France; 3 Radiation Oncology Department, American University of Beirut Medical Center, Beirut, 1107 2020 Lebanon; 4 Département de Radiologie, Centre Léon Bérard, 28 rue Laennec, 69008 Lyon, France; 5 Service de Radiologie, Hôpital d’Instruction des Armées « Desgenettes », Boulevard Pinel, 69003 Lyon, France; 6 ALARA Expertise SAS, 7 Allée de l’Europe, 67960, Entzheim, France

**Keywords:** DNA breaks (double-stranded), Genes (BRCA1/2), Li-Fraumeni syndrome, Neurofibromatosis 1, Radiobiology

## Abstract

**Background:**

While computed tomography (CT) exams are the major cause of medical exposure to ionising radiation, there is increasing evidence that the potential radiation-induced risks must be documented. We investigated the impact of cellular models and individual factor on the deoxyribonucleic acid double-strand breaks (DSB) recognition and repair in human fibroblasts and mammary epithelial cells exposed to current chest CT scan conditions.

**Method:**

Twelve human primary fibroblasts and four primary human mammary epithelial cell lines with different levels of radiosensitivity/susceptibility were exposed to a standard chest CT scan exam using adapted phantoms. Cells were exposed to a single helical irradiation (14.4 mGy) or to a topogram followed, after 1 min, by one single helical examination (1.1 mGy + 14.4 mGy). DSB signalling and repair was assessed through anti-γH2AX and anti-pATM immunofluorescence.

**Results:**

Chest CT scan induced a significant number of γH2AX and pATM foci. The kinetics of both biomarkers were found strongly dependent on the individual factor. The topogram may also influence the biological response of radiosensitive/susceptible fibroblasts to irradiation. Altogether, our findings show that a chest CT scan exam may result in 2 to 3 times more unrepaired DSB in cells from radiosensitive/susceptible patients.

**Conclusions:**

Both individual and tissue factors in the recognition and repair of DSB after current CT scan exams are important. Further investigations are needed to better define the radiosensitivity/susceptibility of individual humans.

**Supplementary Information:**

The online version contains supplementary material available at 10.1186/s41747-022-00266-0.

## Key points

- Chest computed tomography (CT) scan exposure discriminates individuals with double-strand breaks (DSB) as endpoints.
- Cells from *BRCA1*/*BRCA2* mutation carriers elicit more DSB after chest CT scan exposure.
- The justification of CT scans should take into account individual factor.

## Background

To date, computed tomography (CT) scan exams represent the largest cause of medical exposure to ionising radiation (IR). An accurate quantification of the IR-induced cancer risk is therefore becoming a societal, medical, and scientific issue. Furthermore, a reliable biological dosimetry is required to help clinicians in justifying a safe use of diagnostic imaging [1, 2]. While a number of epidemiological studies have been performed to identify the long-term biological consequences of CT scan exposures [3, 4], few studies have raised the question of the influence of individual factor in the biological response to CT scan exposures [5]. In particular, there is evidence that the heterozygous mutations of the *BRCA1* and *BRCA2* genes have been associated with a significant risk of breast cancer [6]. Since the *BRCA1* and *BRCA2* proteins are also involved in the radiation-induced deoxyribonucleic acid (DNA) damage repair and signalling pathway, *BRCA1*^+/−^ and *BRCA2*^+/−^ carriers are considered to be at high risk of IR-induced cancer, notably through chest CT scan exams [5, 7, 8].

Nevertheless, no report has still focused on the biological response of cutaneous fibroblasts and mammary epithelial cells, which are the most relevant cellular models to address this question: the very great majority of studies dealing with the assessment of the DNA damage potentially caused by chest CT scan have been done with *in vitro* or *ex vivo* lymphocytes [8, 9].

IR induces several types of DNA damage, base damage, DNA single-strand breaks, and double-strand breaks (DSB), which differentially participate in the molecular response to IR. Particularly, DSB have been shown to be predictive of radiosensitivity/toxicity if unrepaired, and cellular transformation and radiosusceptibility if misrepaired [10]. While the quantitative correlations between unrepaired DSB and cellular radiosensitivity are well documented at high doses, there is still no consensual correlation between clinical and molecular data for IR-induced cancers.

Recently, a mechanistic model of the response to IR, based on the DSB recognition and repair and on the radiation-induced nucleoshuttling (RIANS) of the ataxia-telangiectasia mutated (ATM) protein kinase was proposed [11–13]. The RIANS model was shown to be relevant for both high- and low-dose exposures [13]. The oxidative stress induced by IR separates cytoplasmic ATM dimers into active monomers. These monomers diffuse to the nucleus and phosphorylate the H2AX histone variant, which reveals DSB sites by the relocalisation of the phosphorylated H2AX (γH2AX) forms as nuclear foci. The ATM-dependent phosphorylation of H2AX and the formation of nuclear γH2AX foci is considered to be the earliest DSB recognition step of the non-homologous end-joining repair pathway, the major DSB repair pathway in humans [13–18]. A delay in RIANS can increase the activity of error-prone DSB repair pathways and favour DSB misrepair through a process called hyper-recombination. The cancer-prone diseases, like those associated with *BRCA1*/*BRCA2* mutations, are systematically associated with hyper-recombination [19]. Hence, by using the formation of nuclear γH2AX and phosphorylated forms of the ATM protein (pATM) foci as endpoints, the risk linked to any exposure to IR can be quantified at the molecular scale [13, 18].

In this study, the response to DSB induced by current chest CT scan exams combined or not with topogram was examined by assessing nuclear γH2AX and pATM foci *in vitro* by using a new generation optical scintillating fibre dosimeter [20], twelve untransformed skin fibroblasts, and four untransformed mammary epithelial cell lines with different radiosensitivity/susceptibility statuses.

## Methods

### Cells

Human untransformed fibroblasts were cultured as monolayers in the conditions detailed elsewhere [11]. The fibroblasts were exposed at passages lower than 15. All the experiments were performed with cells in the plateau phase of growth (95–99% in G0/G1) to overcome any cell cycle effect. Seven fibroblast cell lines were provided from a collection of cells derived from radiosensitive patients, the COPERNIC collection [11]. This collection was approved by the regional ethical committee in respect of the national regulatory procedures. Cell lines were declared under the agreement numbers DC2008-585, DC2011-1437, and DC2021-3957 to the Ministry of Research. The COPERNIC database that gathers radiobiological data of these cell lines was protected under the reference IDDN.FR.001.510017.000.D.P.2014.000.10300. All the anonymous donors were informed and gave signed consent according to the ethics recommendations [11].

Among the COPERNIC cell lines, the 200CLB cell line was derived from an apparently healthy patient and served as the radioresistant control. The 201CLB cell line derived from a *BRCA2*-mutated patient and the 202CLB and 203CLB cell lines derived from *BRCA1*-mutated patients served as representative breast cancer–susceptible examples. All the last three patients underwent a prophylactic mastectomy. The 01HNG, 02HNA, and 13HNG cell lines were derived from patients who showed significant tissue reaction after radiotherapy [11] and served as representative radiosensitive examples. The RACKHAM01, RACKHAM12, and RACKHAM39 cell lines were derived from 3 different neurofibromatosis type 1 *NF1*^*+*/−^ mutated patients. The 85MA cell line was derived from a Li-Fraumeni syndrome (*p53*^*+*/−^ mutated) patient and was a kind gift from D. Scott (Manchester, UK). The GM03399 cell line was purchased from Coriell Institute (Camden, New Jersey, USA) and derived from heterozygous ataxia telangiectasia (*ATM*^*+*/−^ mutated) patients. These last 5 cell lines served as representative non-breast cancer susceptible examples. The origin and the major clinical features of the fibroblast cell lines have been gathered in Table 1.

**Table 1:** Major clinical features of the cell lines used in this study

Cell lines	Cell type	Known gene mutation	Cancer proneness	Radiobiological status
200CLB	Fibroblast	Apparently healthy	nd	Radioresistance
RACKHAM01	Fibroblast	NF1^+/−^	Central and peripheral nervous system tumours	Radiosensitivity and radiosusceptibility
RACKHAM12	Fibroblast	NF1^+/−^	Central and peripheral nervous system tumours	Radiosensitivity and radiosusceptibility
RACKHAM39	Fibroblast	NF1^+/−^	Central and peripheral nervous system tumours	Radiosensitivity and radiosusceptibility
01HNG	Fibroblast	nd (cancer patient)	nd	Radiosensitivity
02HNA	Fibroblast	nd (cancer patient)	nd	Radiosensitivity
13HNG	Fibroblast	nd (cancer patient)	nd	Radiosensitivity
GM03399	Fibroblast	ATM^+/−^	Mainly leukaemia, lymphoma	Radioresistance and radiosusceptibility
85MA	Fibroblast	p53^+/−^	Breast, brain, leukaemia, sarcoma	Radioresistance and radiosusceptibility
201CLB	Fibroblast	BRCA2^+/−^	Breast and/or ovarian cancer	Radioresistance
202CLB	Fibroblast	BRCA1^+/−^	Breast and/or ovarian cancer	Radioresistance
203CLB	Fibroblast	BRCA1^+/−^	Breast and/or ovarian cancer	Radioresistance
201CLBepi	Mammary epithelial cells	BRCA2^+/−^	Breast and/or ovarian cancer	Radiosensitivity and radiosusceptibility
202CLBepi	Mammary epithelial cells	BRCA1^+/−^	Breast and/or ovarian cancer	Radiosensitivity and radiosusceptibility
203CLBepi	Mammary epithelial cells	BRCA1^+/−^	Breast and/or ovarian cancer	Radiosensitivity and radiosusceptibility

*nd* Non-determined

**Fig. 1:**
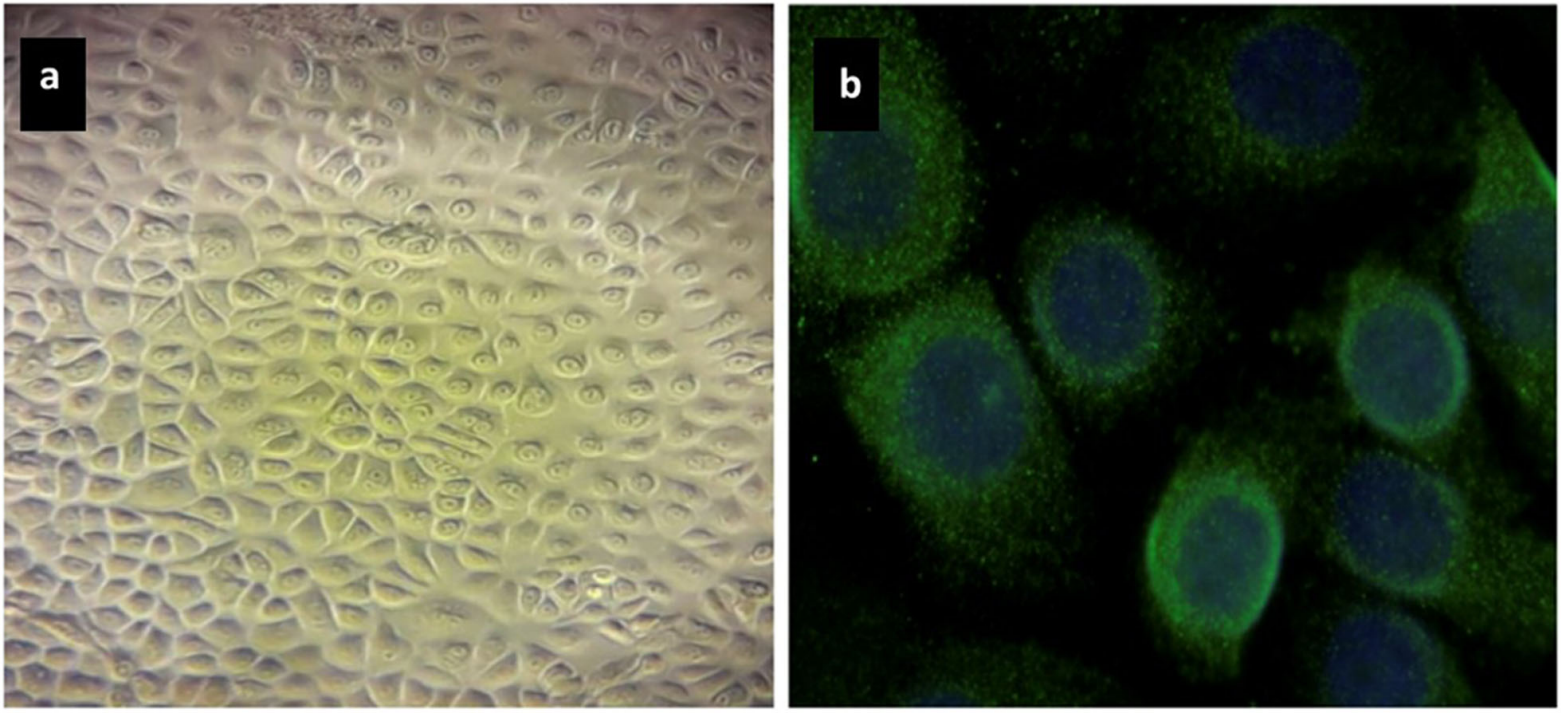
Representative images of mammary epithelial cell culture (**a**) and mammary epithelial cells stained with CK18 to confirm the epithelial nature of cultured cells (**b**)

Four mammary epithelial cell lines derived from the 200CLB, 201CLB, 202CLB, and 203CLB patients were used in this study. These primary mammary epithelial cells were routinely cultured as monolayers with the specific mammary epithelial cell medium MEpiCM provided by Sciencell Research Laboratories (#7611 Sciencell, Carlsbad, USA) supplemented with the specific growth factor complement MEpiCGS (#7652; Sciencell) and penicillin and streptomycin cocktail (#0503; Sciencell) but not fungicide agents. To confirm the epithelial nature of cultured cells, immunofluorescence staining using antibodies against cytokeratin 18 (#ab668; mouse monoclonal CK18 (C-04); dilution 1:100; Abcam SAS, Cambridge, UK) was performed (Fig. 1) [21]. Experiments with mammary epithelial cells were performed at early passages (1 to 4) and at a plateau phase of growth to avoid any bias generated by the cell cycle. The origin and the major clinical features of the 4 mammary epithelial cell lines have been gathered in Table 1.

### Chest CT scan exposure conditions

**Fig. 2:**
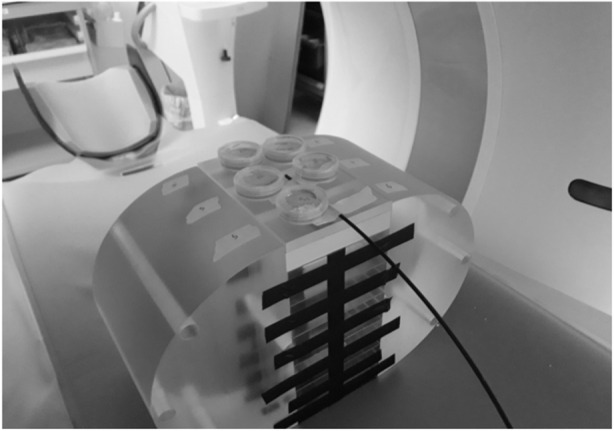
Representative image of the irradiation setup with a polymethyl methacrylate 32-cm width phantom made in an oval shape for a better simulation of the human trunk and the scintillating fibre for dose monitoring

Fibroblasts and mammary epithelial cells were exposed on the phantom surface nearby the 35 × 10-mm petri dishes (#353001; Fisher Scientific, Les Ulis, France), and the absorbed dose was measured with a scintillating fibre dosimeter developed by the Fibermetrix company [20, 22, 23] (Fig. 2). It is however noteworthy that some measures have been done with dosimeter inside (without medium) and nearby the petri dishes and no significant difference has been observed (data not shown). Hence, all the assessments have been performed nearby the petri dishes. Spiral CT scan was performed by using a Somatom Definition Edge scanner (Siemens Healthineers, Erlangen, Germany) operated at 106 to 202 mAs (topogram at 35 mA), 100 kVp, rotation time 0.33 s, pitch 1.2, and collimation 1.5 mm.

### Immunofluorescence analysis

Immunofluorescence protocol for assessing DSB induction and repair was described elsewhere [24, 25]. Briefly, cells were fixed in paraformaldehyde for 15 min at room temperature and permeabilised in detergent solution for 3 min. Primary antibody incubations were performed for 1 h at 37 °C. Anti-γH2AX^ser139^ antibody (#05-636; Merck, Molsheim, France) was used at 1:800, the monoclonal anti-mouse anti-pATM^ser1981^ (#05-740; Merck Molsheim, France) was used at 1:100 and the monoclonal anti-mouse anti-cytokeratin 18 (#ab668; C-04 Abcam SAS, Cambridge, United Kingdom) was used at 1:100. Incubations with anti-mouse fluorescein secondary antibodies provided by Sigma-Aldrich (L’Isle d’Abeau Chesnes, France) were performed at 1:100 at 37 °C for 20 min. Slides were mounted in 4′,6′-diamidino-2-phenyl-indole-stained Vectashield (Vector Laboratories, Burlingame, USA) and cells were counted using a × 100 objective with a fluorescence BX51 microscope (Olympus-France, Rungis, France). For each of the three independent experiments, 100 nuclei were analysed. The patented procedures of foci scoring have been detailed elsewhere [26].

### Data processing and statistics

The data and statistical analyses were processed using MATLAB R2016a (MathWorks, Natick, MA, USA). With regard to the values described in the “Results” chapter, since each experiment is the result of 3 independent replicates with 100 nuclei scored, the mean is given with the standard error of the means (SEM) of the three independent experiments. By contrast, significance tests were performed by grouping the 300 nuclei for each cell line and condition. As a first step, a Kolmogorov-Smirnov test was performed to verify the normality of the distribution of these data in order to choose the appropriate statistical test [27]. The non-parametric Mann-Whitney-Wilcoxon test was used to compare two conditions with each other [28, 29]. When more than two conditions were compared, a Kruskal-Wallis test was performed [30]. For each test, the differences were considered statistically significant when the p-value was lower than 0.05. In the figures, the asterisks shown at the non-irradiated conditions and at 10 min and 1 h post-irradiation times correspond to a significant difference with the radioresistant control data. The asterisks shown at 24 h post-irradiation correspond to a significant difference with the non-irradiated conditions.

## Results

### Radiobiological effects of single helical chest CT scans on cutaneous fibroblasts

Human fibroblasts derived from 12 patients showing different levels of individual radiosensitivity/susceptibility were submitted to one single helical (14.4 mGy) chest CT scan session. This set-up provided an average volumetric CT dose index (CTDIvol) of 8.1 ± 0.6 mGy. The average dose-length product (DLP) was 136.1 ± 11.9 mGy cm. The average absorbed dose at the surface of the phantom was 14.4 ± 1.91 mGy. Concerning the topogram, the CTDIvol was 0.13 ± 0.01 mGy. The average DLP was 6.7 ± 0.6 mGy cm. The average absorbed dose at the surface of the phantom was 1.1 ± 0.06 mGy.

**Fig. 3:**
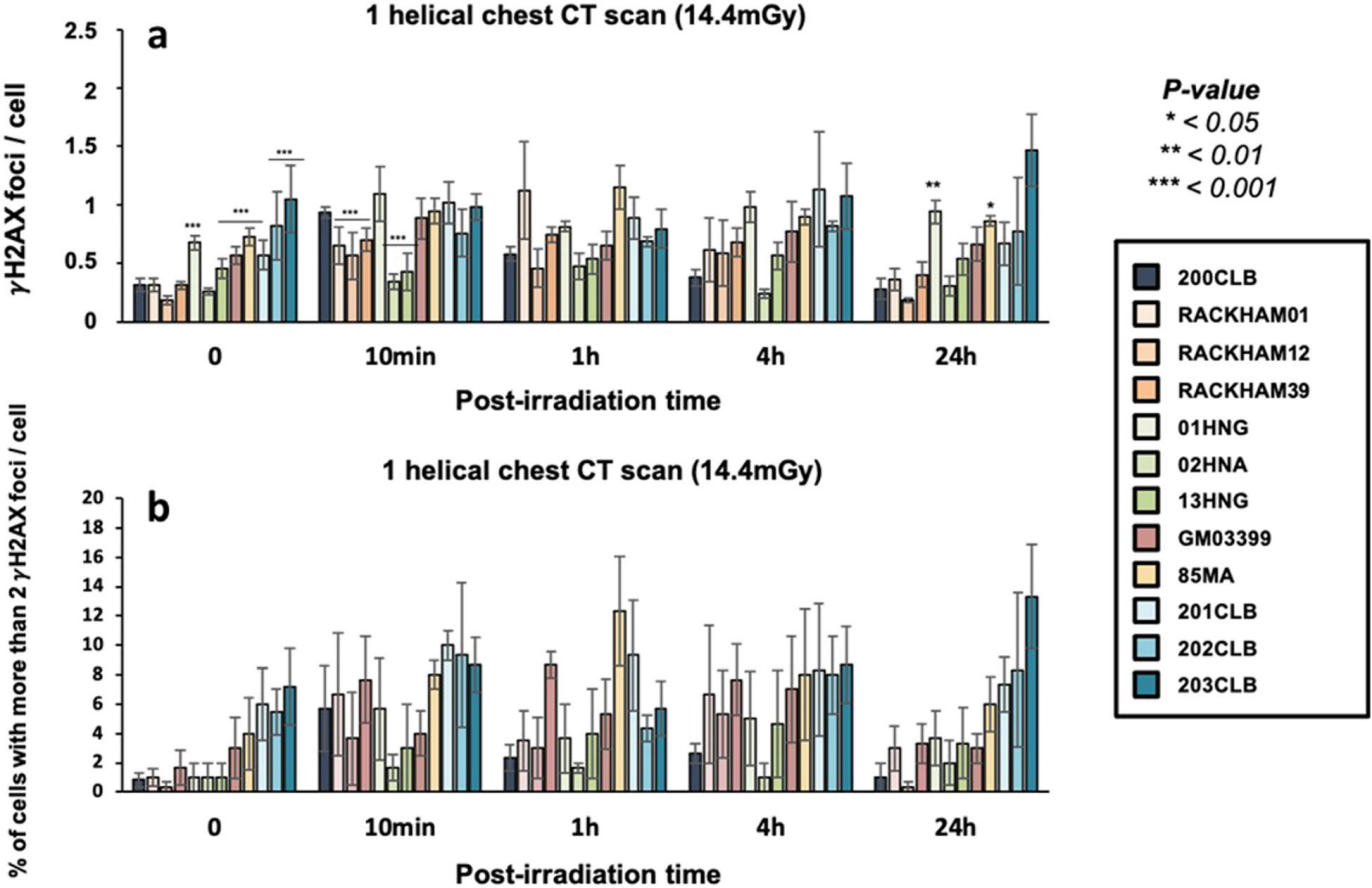
γH2AX foci in fibroblasts after a single helical chest computed tomography scan (**a**). Kinetics of γH2AX foci at the indicated times after exposure (0 = non-irradiated). Each data represents the mean ± standard error of the mean of three independent experiments. The asterisks shown at the non-irradiated conditions and at 10 min and 1 h post-irradiation times correspond to a statistically significant difference with the radioresistant control 200CLB data (*p* < 0.050). The asterisks shown at 24 h post-irradiation correspond to a statistically significant difference with the non-irradiated conditions for the same cell lines (*p* < 0.050). **b** Mean number of cells with more than 2 foci after a single helical chest CT scan. Data is shown as mean ± standard error of the mean of three independent experiments

Without exposure to IR, the radioresistant 200CLB control fibroblasts showed 0.31 ± 0.05 spontaneous γH2AX foci per cell on average. Among the other tested fibroblasts, 6 cell lines (GM03399, 85MA, 01HNG, 13HNG, 201CLB, 203CLB) showed significantly more spontaneous γH2AX foci (*p* < 0.001), suggesting a higher genomic instability. It is however noteworthy that the numbers of γH2AX foci never exceeded 1 focus per cell (Fig. 3a).

Ten minutes after a single helical CT scan exposure, the average number of γH2AX foci was 0.93 ± 0.04 γH2AX foci per cell in the radioresistant controls, corresponding to 0.62 ± 0.05 by omitting the background described above. This value is in agreement with the theoretical rate of DSB induced per Gy per cell currently reported in human diploid fibroblast [25]: a linearly dose-dependent induction of 37 ± 4 γH2AX foci per Gy per cell, corresponds to 0.57 γH2AX foci at 14.4 mGy) (Fig. 3a). Similar conclusions were reached by using the percentage of cells with more than two γH2AX foci as an endpoint (Fig. 3b).

**Fig. 4:**
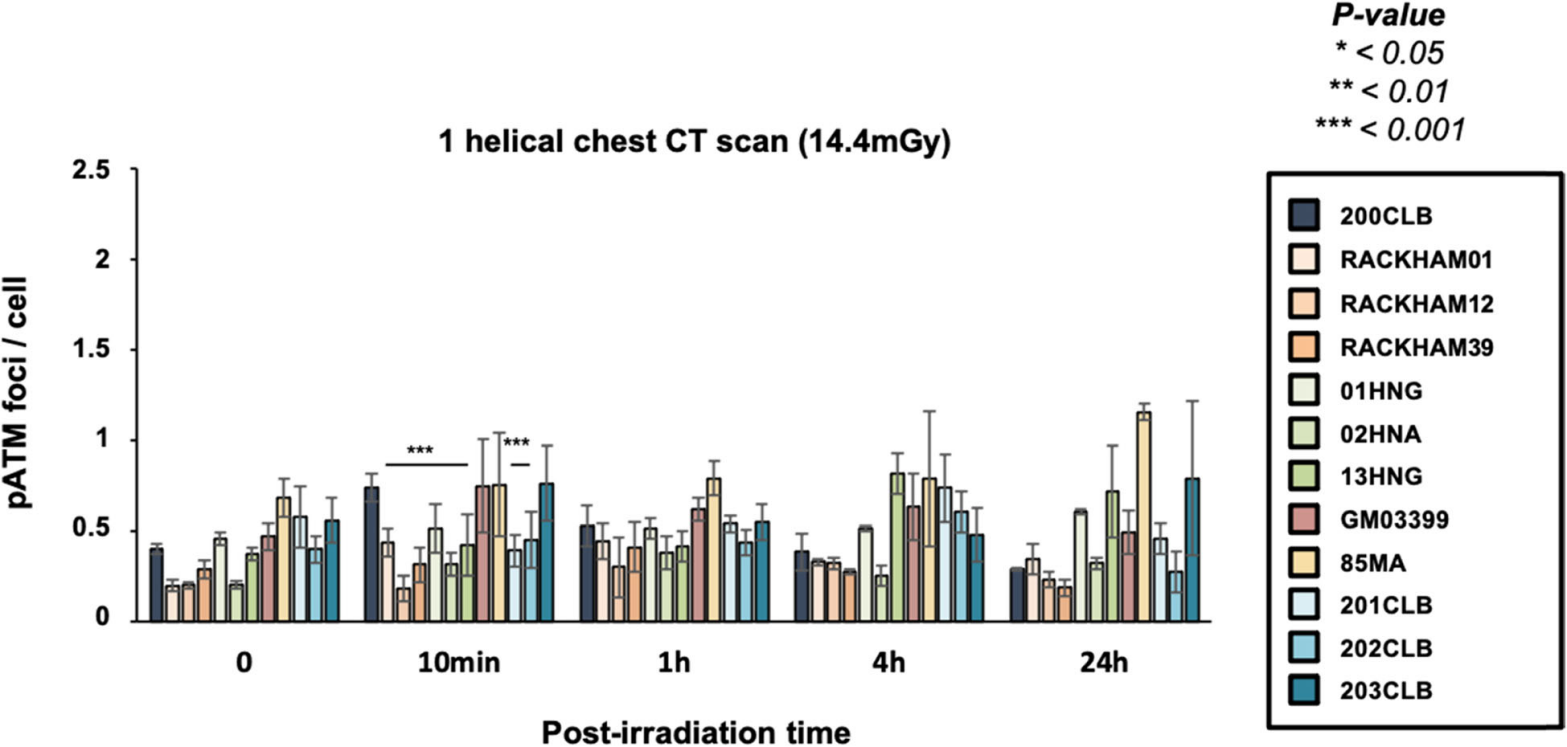
Kinetics of pATM foci in fibroblasts after a single helical chest computed tomography scan. Kinetics of pATM foci at the indicated times after exposure (*t*_0_ = non-irradiated). Each data represents the mean ± standard error of the mean of three independent experiments. The asterisks shown at 10 min post-irradiation times correspond to a statistically significant difference with the 200CLB radioresistant control data (*p* < 0.050)

The average numbers of γH2AX and pATM foci per cell assessed at 10 min after post-irradiation were found significantly lower in RACKHAM01, RACKHAM12, RACKHAM39, 02HNA, and 13HNG cells when compared with data obtained from radioresistant controls (*p* < 0.001) (Fig. 3a and 4). This suggests a less efficient DSB recognition for these cell lines (Supplemental Fig. S1a). In the frame of the RIANS model, these data do not mean that less DSB are *induced* by IR, but rather that less DSB are *recognised* by fewer ATM monomers that diffuse to the nucleus and trigger H2AX phosphorylation (Fig. S1a). In the other cell lines, the early DSB recognition rate was found similar to that of radioresistant controls.

In the radioresistant controls, the number of γH2AX foci significantly decreased with repair time and reached a number of residual γH2AX foci comparable to that assessed in non-irradiated cells. All the other fibroblast cell lines showed a different shape of γH2AX foci kinetic (Fig. 3a and S1a). Particularly, there was a difference in both the maximal number of γH2AX foci and the post-irradiation time at which it was reached. The RACKHAM01, 02HNA, and 85MA fibroblasts reached a maximal number of γH2AX foci at 1 h and the 201CLB fibroblasts at 4 h. For 203CLB, 202CLB, 01HNG, and 13HNG cell lines, the number of γH2AX foci remained constant from 10 min to 24 h post-irradiation, suggesting an impairment in both DSB recognition and repair. All the other cell lines reached their maximal γH2AX value at 10 min post-irradiation and decreased thereafter.

At 24 h post-irradiation, the number of γH2AX foci remaining suggested a complete DSB repair in the radioresistant controls. The 85MA and 01HNG cell lines showed a statistically significant higher number of residual γH2AX foci when compared with non-irradiated conditions (*p* = 0.008 and *p* = 0.001, respectively), suggesting an impairment of DSB repair. All the other cell lines showed a number of residual γH2AX foci similar to that of radioresistant controls, suggesting a normal DSB repair. Again, similar conclusions were reached with the pATM data and with the percentage of cells with more than 2 γH2AX foci (Figs. 3b and 4).

### Radiobiological effects of topogram on cutaneous fibroblasts

**Fig. 5:**
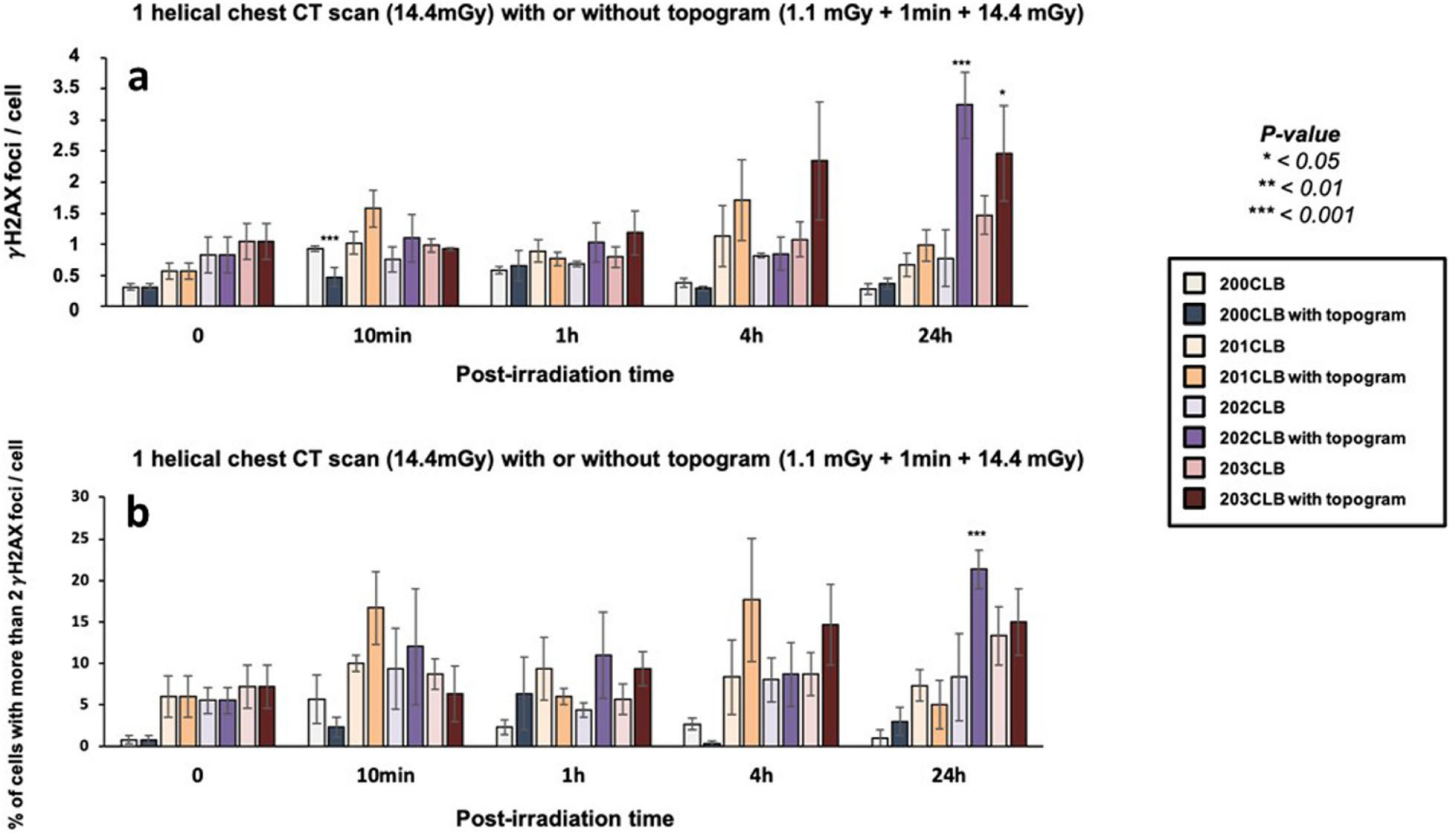
γH2AX foci in fibroblasts after a single helical chest CT scan, with or without topogram. **a** Kinetics of γH2AX foci in fibroblasts after a single helical chest CT scan, with or without topogram at the indicated times after exposure (*t*_0_ = non-irradiated). The irradiation protocol corresponds either to a single dose of 14.4 mGy or to a dose of 1.1 mGy followed, after 1 min, by 14.4 mGy. Each data represents the mean ± standard error of the mean of three independent experiments. Asterisks at 10 min post-irradiation correspond to a statistically significant difference between conditions with or without topogram for the same cell lines (*p* < 0.05)0. The asterisks shown at 24 h post-irradiation correspond to a statistically significant difference with the non-irradiated conditions for the same cell lines (*p* < 0.050). **b** Mean number of cells with more than two foci after a single helical chest CT scan with or without topogram. Data is shown as mean ± standard error of the mean of three independent experiments

The standard protocol of chest CT scan exams generally involves a low-dose topogram, to get a “scout view” of the volume to be imaged. In our conditions, the topogram resulted in a 1.1-mGy dose applied 1 min before the single helical chest CT exposure itself. If the DSB induction rate obeyed a linearly dose-dependent law, a dose of 1 mGy would induce 0.04 DSB per cell, on average, which may be considered negligible. Surprisingly, while this pre-irradiation appeared to have no significant effect in cells up to 4 h post-irradiation, the number of γH2AX foci assessed 24 h post-irradiation was found to be higher than that of non-irradiated cells in the *BRCA1*-mutated cell lines, 203CLB and 202CLB (3.24 ± 0.53 *versus* 0.82 ± 0.29 for 202CLB, respectively; *p* < 0.001 and 2.46 ± 0.77 *versus* 1.05 ± 0.29 for 203CLB, respectively; *p* = 0.040) (Fig. 5a).

**Fig. 6:**
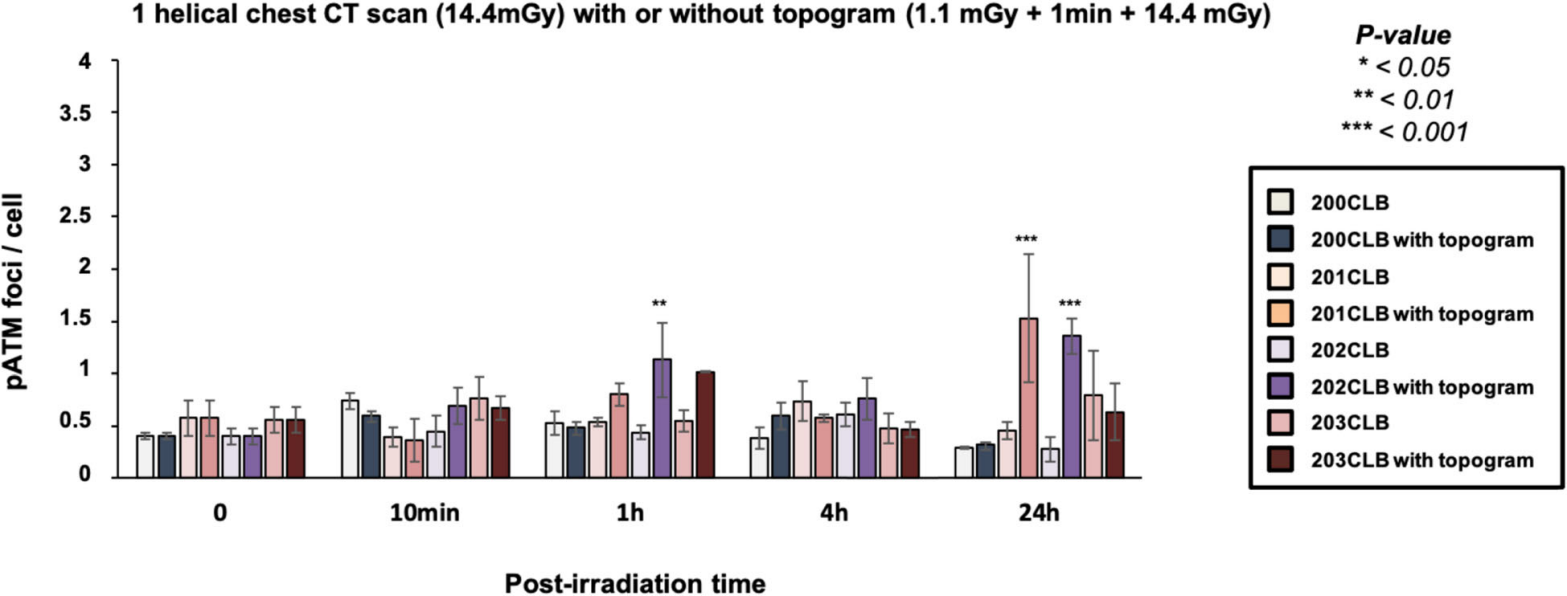
Kinetics of pATM foci in fibroblasts after a single helical chest computed tomography scan, with or without topogram. Kinetics of pATM foci at the indicated times after exposure (*t*_0_ = non-irradiated). The irradiation protocol corresponds either to a single dose of 14.4 mGy or to a dose of 1.1 mGy followed after 1 min, by 14.4 mGy. Data is shown as mean ± standard error of the mean of three independent experiments. Asterisks correspond to a statistically significant difference between conditions with or without topogram for the same cell lines (*p* < 0.050)

When data were expressed as a number of γH2AX foci in excess (Fig. S2a) and as a number of cells with more than two foci γH2AX per cell (Fig. 5b), the same conclusions were reached (Fig. 5b). The pATM data also consolidated our conclusions (Fig. 6 and S2b).

### Radiobiological effects of chest CT on mammary epithelial cells

The same experimental protocol with the same physical features described above was applied to the four mammary epithelial cell lines provided from the 200CLB, 201CLB, 202CLB, and 203CLB donors. In order to avoid any confusion, “epi” labels were added at the end of the name of each mammary epithelial cell line.

**Fig. 7:**
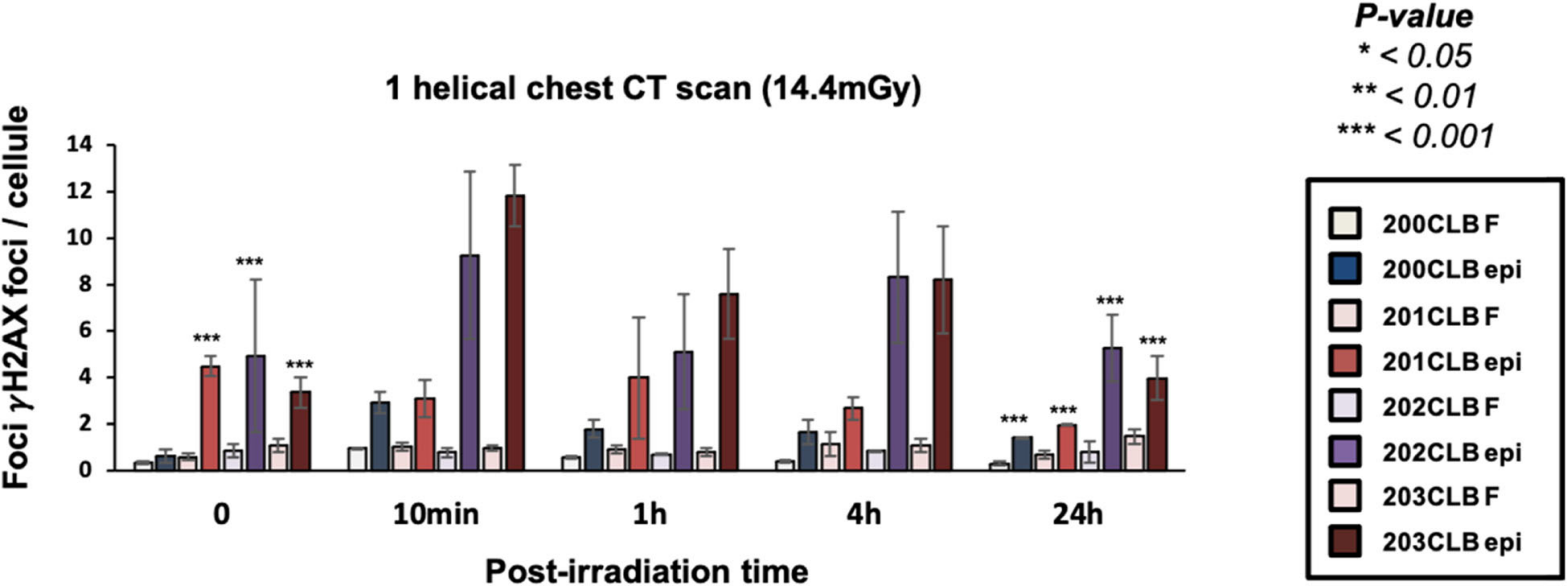
Kinetics of γH2AX foci in fibroblasts (F, light colours) and in mammary epithelial cells (epi, dark colours) after a single helical chest computed tomography scan. Kinetics of γH2AX foci at the indicated times after exposure (*t*_0_ = non-irradiated). Data is shown as mean ± standard error of the mean of three independent experiments. Asterisks at non-irradiated conditions correspond to a statistically significant difference between fibroblasts and mammary epithelial cells from the same patient (*p* < 0.050). The asterisks shown at 24 h post-irradiation correspond to a statistically significant difference with the non-irradiated conditions for the same cell lines (*p* < 0.050)

By considering the spontaneous number of γH2AX foci, no difference was found between the radioresistant 200CLB fibroblast cell line and its corresponding mammary epithelial counterpart, 200CLBepi (0.315 ± 0.053 *versus* 0.6 ± 0.3 γH2AX foci for fibroblasts and mammary epithelial cells, respectively) (Fig. 7). Conversely, for the radiosensitive/susceptible 201CLB, 202CLB, and 203CLB donors, a significant difference was found between the two fibroblastic and mammary epithelial cell types (*p* < 0.001). For example, for the 201CLB cells, there was an 8-fold difference in the number of γH2AX foci between the two cell types (0.57 ± 0.13 *versus* 4.48 ± 0.43 foci for fibroblast and epithelial cells, respectively) (Fig. 7). These findings suggest a strong genomic instability that may be specific to the mammary epithelial cells of these 3 donors.

Ten minutes after a single helical CT scan exposure, a significant increase in the number of γH2AX foci was observed for 200CLBepi, 202CLBepi, and 203CLBepi. These values represented the maximal number of γH2AX foci for these cell lines, suggesting a maximal DSB recognition rate reached early after irradiation (Fig. 7). By contrast, for the 201CLBepi cells, there was no statistically significant increase in the number of γH2AX foci after irradiation. In addition, the number of γH2AX foci at 24 h post-irradiation was lower than that of the non-irradiated controls (*p* < 0.001) (Fig. 7).

The 200CLBepi, 202CLBepi, and 203CLBepi cell lines showed a number of γH2AX foci at 24 h post-irradiation significantly higher than that observed in non-irradiated controls (*p* < 0.001) (Fig. 7).

**Fig. 8:**
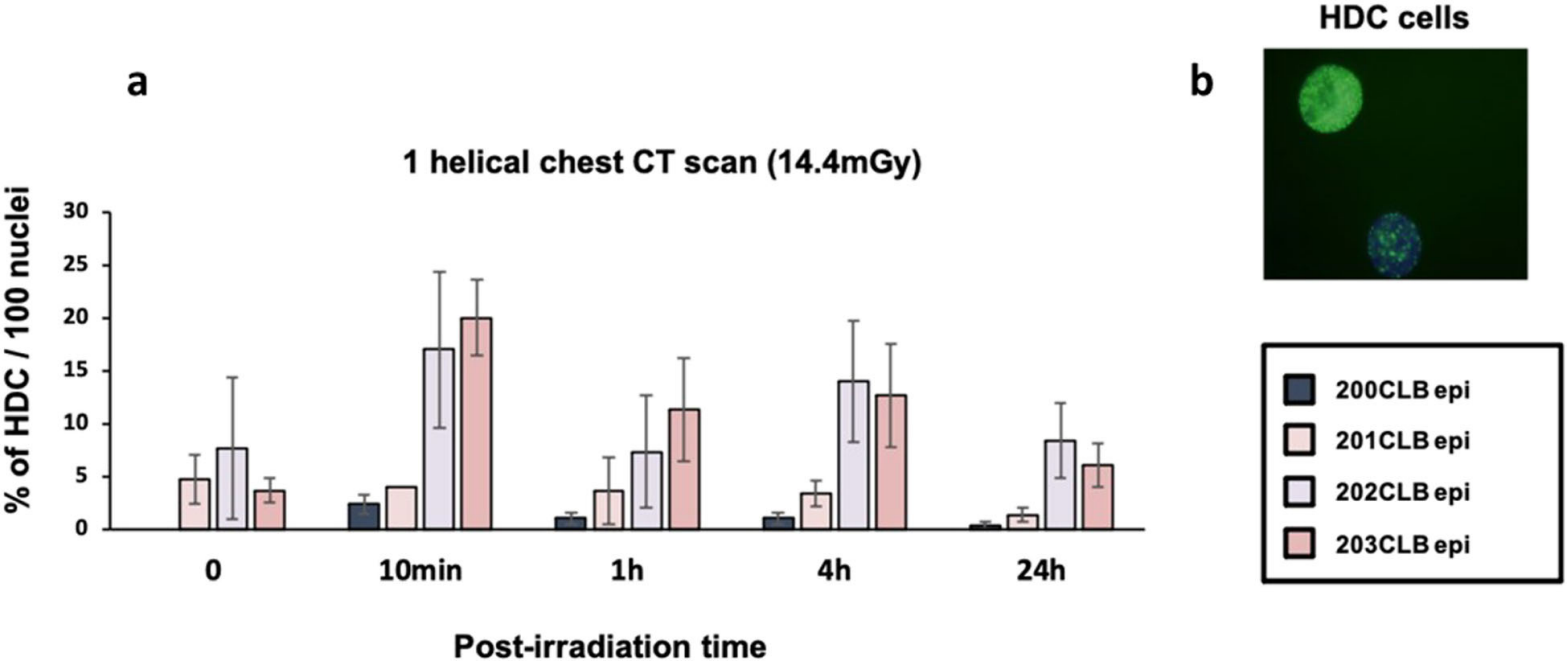
Highly damaged cells (HDC). **a** Percentage of HDC (> 20 foci/cell) in mammary epithelial cells (MEC) after a single helical chest computed tomography scan at the indicated times after irradiation (*t*_0_ = non-irradiated). Data is shown as mean ± standard error of the mean percent of HDC per 100 nuclei of three independent experiments. **b** Representative pattern of HDC with more than 20 γH2AX foci observed in MEC of a *BRCA1* mutation carrier patient

Along our observations, some cells with more than 20 γH2AX foci appeared in certain conditions of exposure (Fig. 8). As already reported, these cells were considered highly damaged cells (HDC) [31]. A relatively small percentage of HDC were found in 200CLBepi and 201CLBepi cell lines, compared to 20% in 202CLBepi and 203CLBepi at 10 min post-irradiation (Fig. 8). The kinetic of the disappearance of HDC roughly followed that of γH2AX foci for each cell line.

The anti-pATM immunofluorescence was also performed on mammary epithelial cells, but the number of pATM foci could not be accurately assessed, mainly because of the predominant cytoplasmic localisation of this protein specific to the epithelial cells (Supplemental Fig. S3).

## Discussion

A number of radiobiological studies have investigated the potential radioinduced risk related to CT scan exposure, but these reports were limited by the use of lymphocytes [8, 9]. Here, for the first time to our knowledge, we exposed skin fibroblasts and primary mammary epithelial cells to current chest CT scan exposure and took into consideration the influence of both the individual radiosensitivity/susceptibility status and the type of tissue to be imaged. In addition, doses were assessed by a new generation optical scintillating fibre dosimeter developed by the Fibermetrix company (Entzheim, France) [20]. The dosimetry indicators generally used in the radiobiological studies involving CT scans, namely CTDIvol and DLP, show many limitations and are not necessarily representative of the dose actually delivered to cells [9, 32]. Hence, our approach allowed us to have more accurate data to provide to the dose-response study. Moreover, given their tightness and their small diameter, these dosimeters permitted to reliably measure the dose on the surface and inside the polymethyl methacrylate phantoms (Fig. 2).

### Delivered doses

According to the report of the French Institute for Radiological Protection [33] concerning CT scan exposures carried out in France in 50 radiology departments, chest CT scans without injection of contrast agent are performed with an average DLP per acquisition of 402 mGy cm, an average CTDIvol of 11.5 mGy and an average number of acquisitions of 1.03. The irradiation conditions applied in this study (DLP 136.1; and CTDIvol 8.1 mGy) are at the lower limit of the dose range of standard chest CT scans [34]. This can be explained by two factors: (1) the use of recent CT scans with effective dose reduction technologies for CTDIvol (Somatom Definition Edge; see Methods); (2) for DLP, the width of the phantom applied in our study (14 cm) was smaller than the area usually explored in clinical condition for a chest CT scan.

In the most current breast CT scan conditions, the dose to the breast was estimated to be between 13 and 20 mGy [34, 35]. Our data were in good agreement with these values (14.4 ± 1.91 mGy). It should also be noted that, although chest CT scans are generally carried out with a single acquisition, some additional acquisitions can be performed, notably if contrast agents are administered [33]. Besides, iodinated contrast agents were also shown to increase the number of unrepaired DSB, which may increase the biological response to exposure [9, 33]. Hence, our protocols are based on the lowest dose currently applied during standard chest CT scan exams. However, despite the absence of iodinated contrast agents, and the low dose (14.4 mGy) applied to cells, a significant number of IR-induced and residual DSB was detected with significant interindividual and intertissue differences.

### Spontaneous DSB

Spontaneous γH2AX foci reflect spontaneous DSB due to exogenous factors (environmental stress and/or radiology history), and/or to impairment in genome maintenance, which is correlated with the hyper-recombination process. This last phenomenon is generally observed in cancer-prone and radiosusceptible patients [31]. The number of spontaneous γH2AX foci per cell was found to be significantly higher in cells from seven patients when compared with the radioresistant control. This observation is in agreement with previous studies focused on the role of ATM, p53, BRCA2, and BRCA1 proteins in genome integrity [36]. This is particularly true regarding the difference of spontaneous γH2AX foci in mammary epithelial cells, inasmuch as the natural tendency of epithelial cells to proliferate may help in increasing and propagating DSB all along the cell cycle.

### Radiation-induced DSB

Our findings suggest that individual factor in skin fibroblasts and mammary epithelial cells can influence the biological responses to current chest CT scan exposures when γH2AX and pATM foci are taken as endpoints. Since these biomarkers reflect the functionality of DSB recognition and repair, our data provide evidence that, even with a single helical CT scan exposure (corresponding here to 14.4 mGy), there is a significant difference between the biological and the physical dose (Fig 3). The number of residual γH2AX foci at 24 h post-irradiation assessed in 01HNG and 85MA cell lines were found to be significantly different after chest CT scan when compared with non-irradiated conditions (01HNG, *p* = 0.001; 85MA, *p* = 0.008). It is noteworthy that the 01HNG cell line is known to be hypersensitive to low doses [37] and the 85MA patient suffered from the Li-Fraumeni syndrome (*p53*^*+*/−^ mutations). All these data are therefore consistent with a significant impact of the individual factor on the final response to IR, even in current chest CT scan conditions. This observation is also confirmed by the high number of DSB observed in mammary epithelial cells (Fig. 7).

### Topogram effect

The effect of the topogram corresponding to a dose delivery pattern of 1.1 mGy followed, after 1 min, by 14.4 mGy. In the frame of the RIANS model, our findings with topogram can be interpreted as the result of two antagonistic phenomena according to the cell lines considered: 1) the additional ATM monomers produced by the topogram may help a higher recognition of DSB; 2) a dose repetition like that induced by the topogram (1.1 mGy + 14.4 mGy) may exacerbate the hyper-recombination process and increases the number of misrepaired DSB. Indeed, the topogram data suggest a dose repetition effect in *BRCA1*-mutated 202CLB and 203CLB fibroblast cell lines (Fig. 5). Indeed, while a small difference in the number of γH2AX foci was assessed between the radioresistant controls and the two *BRCA1*-mutated cells with a single helical CT scan exposure, this difference became significant (*p* < 0.001 and *p* = 0.040, respectively) when a topogram was added to the irradiation protocol. Previous studies have reported a similar phenomenon after two successive low doses mimicking a two-view mammography screening (2 mGy, followed after 3 min, by 2mGy) [38]. In our previous study about mammography, the impact of the time between the two irradiations was highlighted, showing that this delay was not enough to allow a full DNA breaks recognition and repair. As a consequence, chromatin is more decondensed when the second dose is delivered, which favours the induction of additional DSB and illustrate well the potential role of single-strand breaks. This effect is called the “low and repeated dose”, LORD, effect [38].

When radiosusceptible cells were subjected to these specific irradiation conditions, the hyper-recombination process was shown to be exacerbated. This trend was notably observed by the increase in HDC cells. This effect producing additional endogenous DNA breaks was called the “low-dose additional and dose-induced”, LADI, effect and was found specific to cells from cancer-prone patients [38]. Interestingly, in this study, a LADI effect and HDC were observed in mammary epithelial cells provided from *BRCA1*-mutated patients. Interestingly, the 4', 6'-diamidino-2-phenyl-indole-staining indicated that the morphological shape of HDC was clearly different from that of apoptotic cells. The increase in HDC yields cannot correspond to the proportion of cells in the *S* phase since the percentage of HDC increased with dose. A more plausible interpretation would be that HDC contain several misrepaired DSB that, despite their number, do not lead to cell death. Again, this interpretation is consistent with the fact that there were more HDC in *BRCA1*-mutated cells.

At any rate, further investigations are needed to better understand the consequences of such low-dose repetition effects and the potential link between HDC, cellular lethality, and carcinogenesis, notably in patients whose cells undergo hyper-recombination.

## Conclusions

The present study was performed by applying current chest CT scan conditions to relevant cellular models, *i.e*., cutaneous fibroblasts and mammary epithelial cells. Dosimetric monitoring was carried out with a new technology using a small waterproof scintillating fibre. These dosimeters allowed accurate measurement of the dose, providing a new useful tool for radiobiologists. Our data showed that the radiation response is influenced by the presence of genetic mutations associated with radiosensitivity and/or radiosusceptibility [10]. Even if DSB misrepair, hyper-recombination, and genomic instability are systematically associated with cancer proneness, a reliable biomarker specific to radiosusceptibility and applicable in CT scan conditions is required.

The RIANS model based on the nucleoshuttling of the ATM protein after irradiation was already shown to be relevant at a low dose and proposes a mechanistic interpretation of both radiosensitivity and radiosusceptibility [12, 39, 40]. Here, by using RIANS biomarkers, this study strongly suggests that, even for a relatively low dose (14.4 mGy), the number of recognised and/or residual DSB significantly differs from a patient to another.

Altogether, these data provide an additional proof that the justification of the CT scan exam should take into account the individual factor. Additional studies are however needed to reliably quantify the risk for a given genetic status.

## Supplementary Information

**Additional file 1.** Electronic Supplementary Material.

## Data Availability

The data presented here are present in a deposed database (see Methods) and will be made available on reasonable request.

## Abbreviations

ATM: Ataxia telangiectasia mutated gene/protein
BRCA1/BRCA2: Breast cancer susceptibility gene/protein ½
CT: Computed tomography
CTDIvol: Volumetric CT dose index
DLP: Dose-length product
DNA: Deoxyribonucleic acid
DSB: DNA double-strand breaks
HDC: Highly damaged cells
IR: Ionising radiation
pATM: Phosphorylated forms of the ATM protein
RIANS: Radiation-induced nucleoshuttling of the ATM protein
γH2AX: Phosphorylated forms of the H2AX histone variant molecular
