# Supplementary material for: Influence of cellular models and individual factor in the biological response to chest CT scan exams
Source: Eur Radiol Exp. 2022 Mar 17;6:14. doi: 10.1186/s41747-022-00266-0 (PMC8931147; doi:10.1186/s41747-022-00266-0)
Supplement: Supplementary file 1 — Additional file 1. Electronic Supplementary Material. [file 41747_2022_266_MOESM1_ESM.docx]

**ELECTRONIC SUPPLEMENTARY MATERIAL**

**Influence of cellular models and individual factor in the biological response to chest CT scan exams**


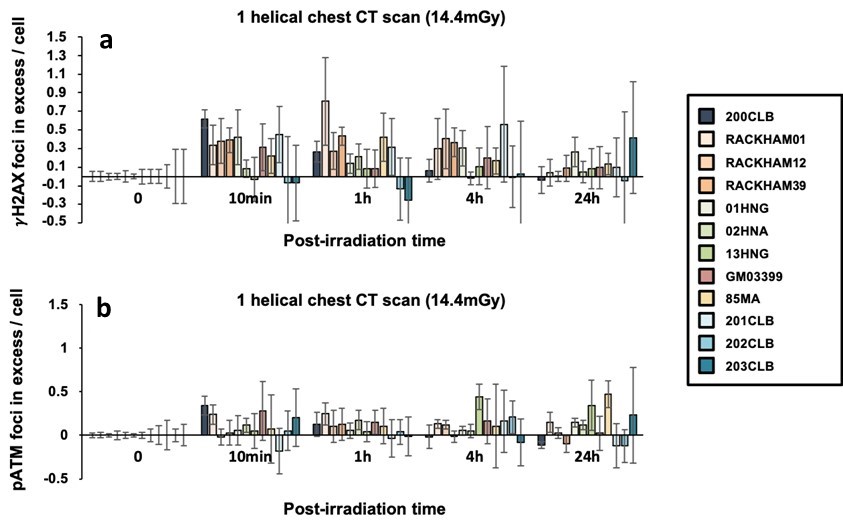


**Figure S1: Kinetics of γH2AX and pATM foci in excess after a single helical Chest CT. (a)** Kinetics of γH2AX foci and **(b)** pATM foci in excess in fibroblasts after a chest CT scan at the indicated post-irradiation times (t0 = non irradiated). Data result from those shown in Figure 6 with background subtraction in order to show γH2AX foci in excess effectively due to CT exposure. Error bars indicate SEM.

**Figure S2:**


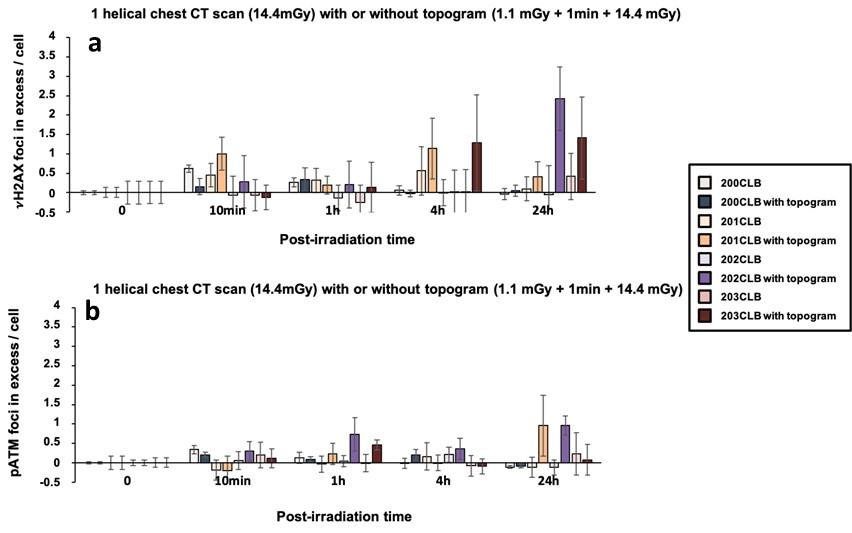


**Figure S2: Kinetics of γH2AX and pATM foci after a single helical chest CT with or without topogram. (a)** Kinetics of γH2AX foci and **(b)** pATM foci in excess in fibroblasts after a chest CT scan with or without topogram at the indicated post-irradiation times (t0 = non irradiated). Data result from those shown in Figure 6 with background subtraction in order to show γH2AX foci in excess effectively due to CT exposure. Error bars indicate SEM.

Figure S3:


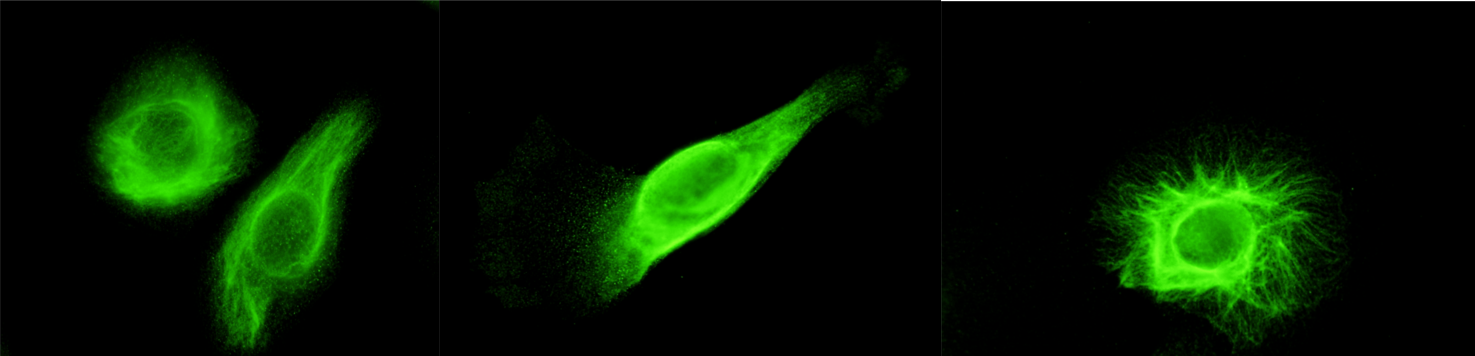


**Figure S3: Representative examples of** **mammary epithelial cells pATM staining.** This staining was generally found cytoplasmic whatever the experimental conditions and did not allow us to reliably count the number of pATM foci per cell when they were present in nucleus.
